# Decoding the host–microbiome dialogue with biological foundation models

**DOI:** 10.1016/j.ebiom.2026.106394

**Published:** 2026-07-08

**Authors:** eBioMedicine

Why do microbiome-based therapies so often fail to reproduce their preclinical promise in patients? A recent review (*Gut*, June, 2026) noted that, beyond faecal microbiota transplantation for recurrent *Clostridioides difficile* infection, microbiome-targeted interventions have yet to meet their high expectations, underscoring a persistent translational bottleneck. Microbiome research has been far more successful at identifying associations than at determining mechanistic microbial functions. Artificial intelligence (AI) might offer a route forward.

The microbiome is not merely a catalogue of species but a large signalling system whose effects are mediated by metabolites, cell-surface structures, secreted proteins, and small peptides. Yet many genes encoding these molecules remain uncharacterised—often referred to as the functional microbial dark matter the functional microbial dark matter. The central question is therefore no longer simply which microbes are present, but which microbial functions are active, and how host genetics, immune context, and timing shape their influence.

As outlined in a *Lancet Microbe* review (May, 2026), AI could help moving microbiome research from descriptive community profiling towards predictive modelling. Biological foundation models offer one way to decipher this hidden layer. Just as large language models learn patterns in human language, these models learn representations from protein or genomic sequences. Researchers can now move beyond classical homology and infer what a microbial protein might do from statistical regularities embedded in its sequence. Evo2 (*Nature,* March, 2026), a genomic foundation model trained on trillions of nucleotides across all domains of life, illustrated how such approaches can predict functional properties at scale. More targeted peptide models published by Cheng and colleagues (*Nature Communications*, August, 2024) and Hou and colleagues (*Science Advances*, March, 2025) have moved towards antimicrobial-peptide design, generating candidates active against multidrug-resistant pathogens in mouse models.

Understanding the intersection between the microbiome and host immunity is another compelling application of AI-assisted approaches. In a 2025 *eBioMedicine* study, Ma and colleagues screened over 300,000 human microbial genomes for peptides predicted to mimic the myelin oligodendrocyte glycoprotein (MOG), a central nervous system antigen targeted by T cells in autoimmune demyelinating disease. By combining AlphaFold-based modelling with peptide-MHC-TCR docking, the authors identified candidate microbial mimics. Among these mimics, P3 was capable of activating MOG-specific CD4^+^ T cells and increasing autoimmune neuroinflammation in mice. This finding provides a mechanistic example of how previously uncharacterised microbial peptides can shape host immune responses. Such candidate-level predictions become more informative when placed in genomic context. A genomic language model (*Nature Communications*, April, 2024) trained on millions of metagenomic scaffolds learned functional relationships and operon-level co-regulation among neighbouring genes.

Yet, enthusiasm for such models must be treated with caution. Benchmarking efforts (*Nature Communications*, November, 2025) found that the performance of DNA foundation models was highly task-dependent, with clear trade-offs. Although foundation models could outperform conventional convolutional neural networks in some genome classification tasks, they underperformed in multispecies and epigenetic predictions where task-specific optimisation is required. Such limitations may be particularly consequential in microbiome research, where contamination, low biomass, and uneven sampling remain challenges.

Moreover, host genetics adds another layer of complexity. In a cohort study, Ludvigsson and colleagues (*Nature Communications*, August, 2019) showed that HLA class II alleles are associated with microbial alterations and autoimmune risk. Moreover, Xavier and colleagues (*Immunity*, October, 2022) showed that microbial peptides elicit different T-cell responses depending on the host genetic background and immune context. Although not foundation-model studies, this research highlights the profound host heterogeneity that biological foundation models must eventually capture if they aim to predict host–microbe interactions with clinical relevance.

Bridging the gap between *in silico* prediction and biological relevance also requires new experimental systems. Engineered microbes, including probiotic platforms, offer one direction. Wang and colleagues (*Science Advances*, November, 2025) showed that a probiotic engineered to release a GLP-1-boosting peptide improved gut function and reduced colitis in mice. Such engineered microbes could serve as testbeds for evaluating AI-designed peptides directly within host tissues. Organoid microbiome co-cultures, spatial immune profiling and high-throughput antigen screening will likewise be essential for anchoring computational predictions in biological reality.

Future biological foundation models will require thoughtful integration with experimental validation, consideration of host heterogeneity, and clear reporting standards. The goal is not merely to generate hypotheses, but to decipher the molecular crosstalk of microbial activity with host immunity and physiology. At *eBioMedicine*, we encourage studies that integrate foundational models with robust validation in human, animal, or organoid systems—especially studies going beyond association to show how particular microbial signals are interpreted and acted upon by the host.

